# Behavioral interactions of bed bugs with long-lasting pyrethroid-treated bed nets: challenges for vector control

**DOI:** 10.1186/s13071-022-05613-z

**Published:** 2022-12-26

**Authors:** Christopher C. Hayes, Coby Schal

**Affiliations:** Department of Entomology and Plant Pathology, North Carolina State University, Raleigh, NC USA

**Keywords:** *Cimex lectularius*, Malaria, Long-lasting insecticide nets, Resistance evolution, Insect behavior

## Abstract

**Background:**

Widespread vector control has been essential in reducing the global incidence and prevalence of malaria, despite now stalled progress. Long-lasting insecticide-treated nets (LLINs) have historically been, and remain, one of the most commonly used vector control tools in the campaign against malaria. LLINs are effective only with proper use, adherence, retention and community adoption, which historically have relied on the successful control of secondary pests, including bed bugs. The emergence of pyrethroid-resistant bed bugs in malaria-endemic communities and failure to control infestations have been suggested to interfere with the effective use of LLINs. Therefore, the behavioral interactions of bed bugs with commonly used bed nets should be better understood.

**Methods:**

To investigate the interactions between bed bugs (*Cimex lectularius* L.) and LLINs, insecticide-susceptible and pyrethroid-resistant bed bugs were challenged to pass through two commonly used LLINs in two behavioral assays, namely host (blood meal)-seeking and aggregation-seeking assays. The proportions blood-fed and aggregated bed bugs, aggregation time and mortality were quantified and analyzed in different bed bug life stages.

**Results:**

Overall, both the insecticide-susceptible bed bugs and highly resistant bed bugs showed a varying ability to pass through LLINs based on treatment status and net design. Deltamethrin-treated nets significantly impeded both feeding and aggregation by the susceptible bed bugs. While none of the tested LLINs significantly impeded feeding (passage of unfed bed bugs through the nets) of the pyrethroid-resistant bed bugs, the untreated bed net, which has small mesh holes, impeded passage of fed bed bugs. Mortality was only seen in the susceptible bed bugs, with significantly higher mortality on deltamethrin-treated nets (63.5 ± 10.7%) than on permethrin-treated nets (2.0 ± 0.9%).

**Conclusions:**

Commonly used new LLINs failed to prevent the passage of susceptible and pyrethroid-resistant bed bugs in host- and aggregation-seeking bioassays. The overall low and variable mortality observed in susceptible bed bugs during both assays highlighted the potential of LLINs to impose strong selection pressure for the evolution of pyrethroid resistance. Already, the failure to control bed bug infestations has been implicated as a contributing factor to the abandonment or misuse of LLINs. For the first time to our knowledge, we have shown the potential of LLINs in selecting for resistant secondary pest populations and so their potential role in stalling malaria control programs should be further investigated.

**Graphical Abstract:**

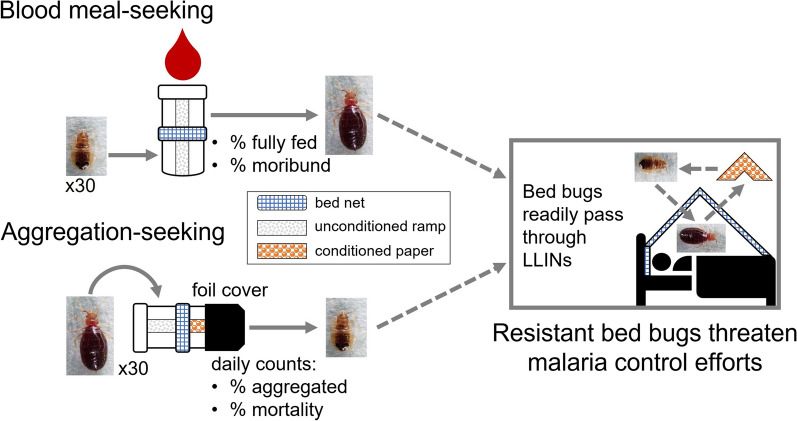
The emergence of pyrethroid-resistant bed bugs in malaria-endemic communities may interfere with the effective use of pyrethroid-impregnated bed nets. We assessed the interactions of two bed bug strains with commonly used bed nets using two behavioral assays, namely host (blood meal)-seeking by unfed bed bugs and aggregation-seeking by freshly fed bed bugs. These assays assessed the passage of bed bugs through various bed nets in response to host cues and aggregation stimuli, respectively. Conditioned paper is a section of file folder paper that has been exposed to bed bugs and has been impregnated with feces and aggregation pheromone; it is attractive to aggregation-seeking fed bed bugs. An unconditioned ramp is a similar section of file folder paper that allows bed bugs to traverse the bed net and gain access to a blood-meal source.

## Background

### Malaria and the role of bed nets in malaria control

Vector-borne diseases (VBDs), those diseases that are transmitted through the bite of an infected arthropod vector, threaten nearly 82% of the global population and disproportionately affect those living in the poorest areas [1]. Malaria, a parasitic protozoan disease vectored to humans through the bite of an infected *Anopheles* mosquito, is the most widely studied VBD and remains one of the most severe public health problems worldwide [2]. Efforts to reduce the prevalence and incidence of malaria have historically been relatively successful [3]. Despite these advances, however, recent research has shown that malaria remains endemic in all World Health Organization (WHO) regions and that substantial previous gains in malaria control have slowed [4]. Today, nearly half the world’s population lives in malaria-endemic areas. Accurate surveillance of disease incidence and malaria control presents a major challenge in some countries while others are moving closer to malaria elimination [5].

Mosquito control has been indispensable in reducing both the incidence and prevalence of malaria globally, being alone responsible for a roughly 33% decrease in malaria burden across Africa from 2000 to 2010 [6–8]. Commonly used malaria vector control practices include environmental management to remove oviposition sites, larvicide use, the application of residual insecticides to interior surfaces of homes known as indoor residual sprays (IRS), area-wide spraying, the use of novel baits and the widespread deployment of pyrethroid-treated bed nets known as long-lasting insecticide-treated nets (LLINs) [9, 10]. LLINs are the most widely distributed and used method of vector control due to their effectiveness at both repelling and killing mosquitoes indoors and thus reducing biting rates, low cost, sustainability and reliability to reduce the burden of malaria transmission across a varied epidemiological landscape [11]. Adherence to bed net use and their retention, both of which rely heavily on community perception and commitment, are key to effective vector control, reducing biting rates and the successful reduction and elimination of malaria from endemic regions.

### Impact of secondary pests on vector control

Homes in low- and low-middle income countries, where vector control measures are commonly deployed indoors, are often infested with other hematophagous pests, such as kissing bugs, fleas, lice and, most notably, bed bugs. Unlike mosquitoes, all mobile life stages of bed bugs are hematophagous and flightless, and therefore they live in close proximity to humans. Whereas *Cimex lectularius* is primarily distributed in temperate regions, *Cimex hemipterus* is found primarily in tropical regions [12, 13]; however, populations of both species overlap and have been shown to co-infest dwellings in communities across Africa, Asia, Australia, North America and more recently Europe, mainly through global travel [14–18]. Propagules of just a few bed bugs can quickly expand into large infestations in the absence of effective control measures because of their tolerance of inbreeding, relatively high fecundity, rapid development, long adult life and cryptic nature [19]. Bed bugs are generally considered a nuisance pest, as they are not known to vector any pathogens to their human hosts. However, their bites have the potential to cause severe allergic reactions, painful inflammation, itching as well as anemia in rare cases associated with severe infestation [20]. Additionally, bed bug feces contain large amounts of histamine, a multi-functional immune modulator and neurotransmitter known to cause muscle swelling, itching and other allergic responses, further highlighting the potential harmful effects associated with bed bug infestations [21]. Therefore, effective control of bed bug infestations is a priority for residents globally, regardless of income status.

Many communities that use LLINs prefer to focus on “practical or secular concerns” such as the control of indoor urban pests rather than the long-term national and generational health benefits associated with decreased disease transmission and vector control [22]. Thus, in resource-limited malaria-endemic communities, where bed bug control is unaffordable or unavailable, LLINs are heavily relied upon for bed bug control [23]. As a result, the control of bed bugs can directly impact bed net uptake and adherence to their proper use [22]. This effect was shown in the late 1990s in Tanzanian communities where, for example, > 50% of sampled homes reported bed bug infestations, and the perceived suppression of bed bugs by LLINs led to significantly greater net uptake and retreatment rates as well as greater adherence to bed net use [24]. By the early 2000s, however, anti-malaria campaigns suggested that dichlorodiphenyltrichloroethane (DDT)-resistant bed bugs impeded malaria control efforts through disruption of community adherence, leading to refusal of DDT treatments [25]. Even now, communities in sub-Saharan Africa continue to rate the effectiveness of distributed LLINs on their ability to kill secondary pests around their homes, including house flies, fleas, cockroaches and bed bugs; low efficacy of LLINs on secondary pests can result in net abandonment and misuse [26–29]. Additionally, widespread bed bug infestations in Rwandan communities despite LLIN interventions were a “major hindrance” to the continued effective use of these vector control tools [30]. These studies highlight the intricate connection between LLIN use for vector control in malaria-endemic communities and collateral effects, namely the impact of LLINs on secondary indoor pests. Emerging evidence suggests that inefficacy of LLINs in the control of secondary indoor pests, especially bed bugs, can significantly disrupt LLIN adoption and their sustained use.

### Insecticide resistance in bed bugs may disrupt LLIN use

Insecticide resistance is a major impediment to timely suppression and eradication of bed bug populations [31]. Bed bugs have a long history of evolving and maintaining resistance to insecticides, with the first major example being resistance to DDT observed in 1947, which persists in populations today [32, 33]. Modern examples include resistance to other organochlorine insecticides, carbamates, organophosphates, pyrethroids, neonicotinoids and fipronil (phenylpyrazole; not labeled for the control of bed bugs) [33, 34]. These patterns demonstrate that bed bugs rapidly evolve adaptive responses to insecticides that target them. Moreover, the unintentional exposure of bed bugs to insecticides not labeled for their control may impose strong selection pressure and lead to the evolution of insecticide resistance. LLINs may similarly expose bed bugs to pyrethroid insecticides. Indeed, recent research has shown that exposure of bed bugs to common LLINs in the field resulted in low mortality even in homes where no prior chemical control specifically targeted against bed bug was reported [23].

Selection by LLINs for pyrethroid resistance in bed bug populations may disrupt LLIN-based malaria control efforts. Behavioral interactions of bed bugs with LLINs are therefore the likely setting where selection for pyrethroid resistance occurs. To our knowledge, however, there are no studies of bed bug-bed net interactions. Therefore, we designed behavioral assays that required host-seeking and aggregation-seeking bed bugs (*C. lectularius*) to interact with and pass through pyrethroid-treated bed nets and insecticide-free bed nets. It is imperative to discern what role, if any, LLINs may play in influencing or disrupting these two behaviors. Understanding these interactions may mitigate barriers to LLIN use and guide the development and implementation of more effective bed nets to achieve malaria elimination while also suppressing bed bug infestations.

## Methods

### Colony maintenance and feeding

Two laboratory-maintained strains of *C. lectularius* were used in this study. The Harlan strain (also known as Ft. Dix) is an insecticide-susceptible strain commonly used as a reference. It was collected at Fort Dix, New Jersey, USA, in 1973 and has not been challenged with insecticides since collection. The Harlan strain was maintained on a human host until December 2008, then on defibrinated rabbit blood until July 2021 and on human blood thereafter. The Fuller Mill strain was collected in a residence in High Point, North Carolina, USA, in 2017. Compared to the Harlan strain, the Fuller Mill strain has ~ 1000-fold resistance to permethrin (Hayes and Schal, unpublished) and 44.4-fold resistance to fipronil [34]. Both bed bug colonies were maintained at 35–45% relative humidity, 25 °C, on a photoperiod of 12:12 (light:dark) h, and fed weekly on heparinized human blood (supplied by the American Red Cross under IRB #00000288 and protocol #2018-026). We used an artificial feeding system, which has been previously described [35]. The feeding system was housed in a North Carolina State University-approved BSL-2 facility (Biological Use Authorization #2020-09-836). Between feeding sessions, the glass feeders were sanitized with 7.5% sodium hypochlorite and 95% ethanol and air-dried.

### Bioassay apparatus design

**Fig. 1:**
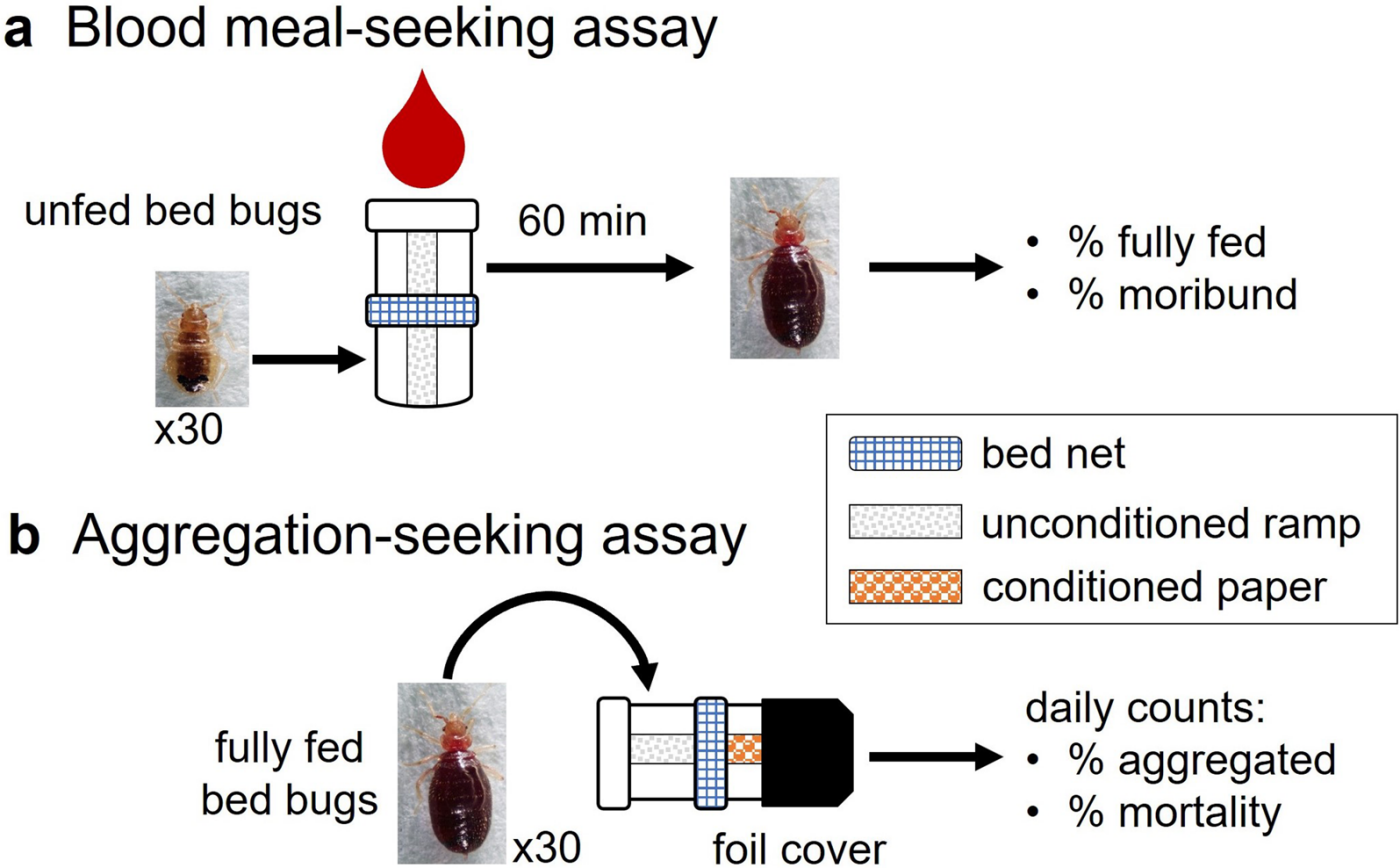
Graphical representation of two behavioral assays designed to assess the interactions of two *C. lectularius* strains with commonly used bed nets. The bioassays quantified two distinct bed bug behaviors, namely blood meal (host)-seeking behavior of unfed bed bugs (**a**) and aggregation-seeking behavior of freshly fed bed bugs (**b**). These assays assessed the passage of bed bugs through various bed nets in response to host cues and aggregation stimuli, respectively. Note that the aluminum foil in **b** completely covers the aggregation jar (see Fig. 2c) but is truncated in this illustration to show the conditioned (feces- and aggregation pheromone-impregnated) section of file folder paper within the jar. The unconditioned ramps are sections of file folder paper that allow bed bugs to reach and traverse the bed net to gain access to a blood-meal source

**Fig. 2:**
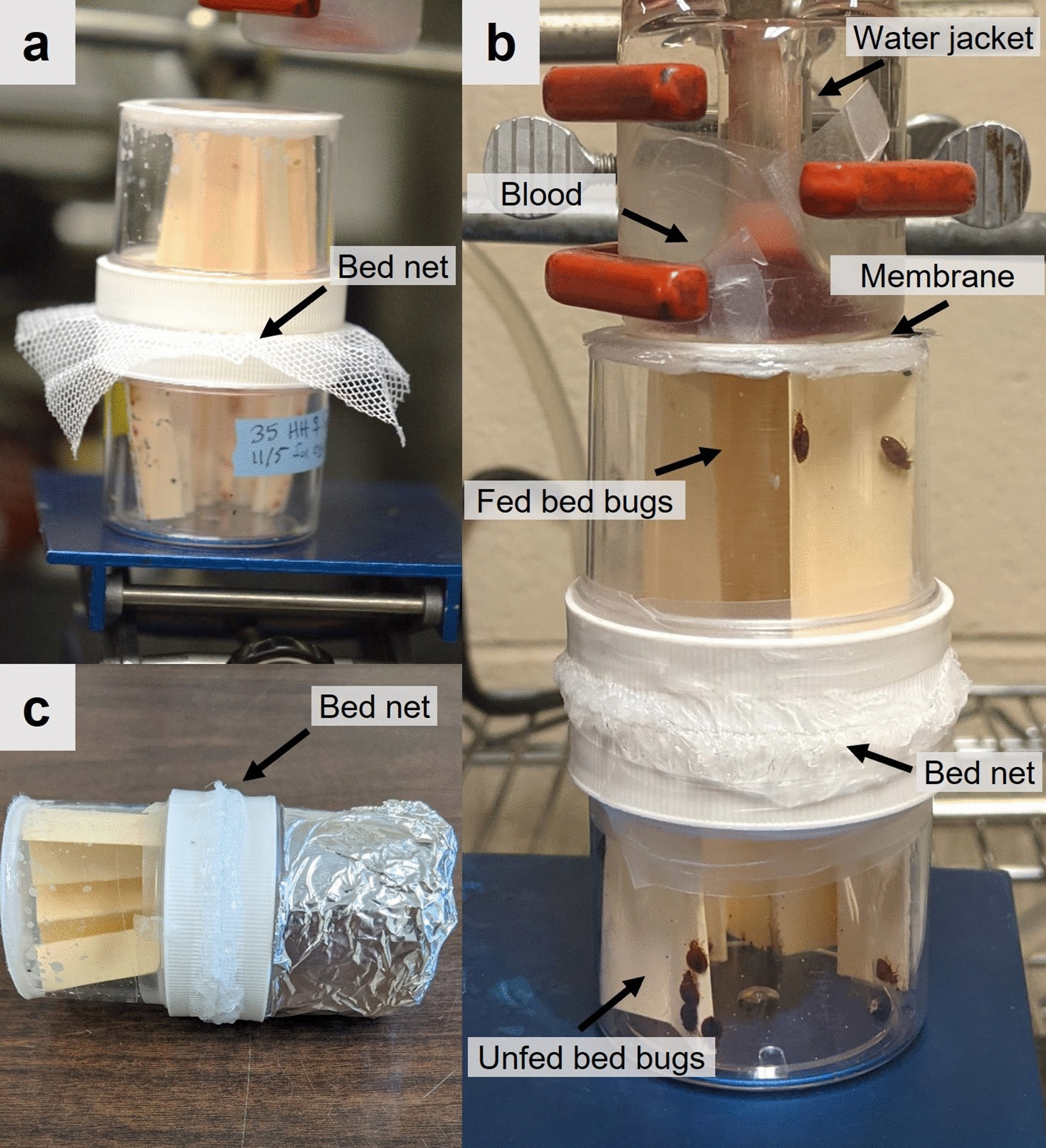
Photos showing the two-jar bed net assembly used in the two behavioral assays. The two-jar assembly before the sandwiched bed net was trimmed (**a**). The blood meal-seeking assay in its vertical orientation, showing unfed *C. lectularius* at the starting point in the bottom jar and fed bed bugs in the top jar that abuts the blood feeder (**b**). The aggregation-seeking assay in the horizontal orientation, with fed bed bugs placed in the exposed jar (left) and the target aggregation jar (right) containing a section of conditioned (feces- and aggregation pheromone-laden) file folder paper and darkened with aluminum foil (**c**)

We designed and validated two bioassays that assessed the passage of bed bugs through various bed nets. The first assay, the blood meal-seeking assay, quantified the passage of unfed, host-seeking bed bugs through bed nets. This assay was designed on the premise that unfed bed bugs are highly motivated to seek a blood meal and are highly responsive to host-emitted stimuli [36]. The second assay, the aggregation-seeking assay, quantified the passage of fully fed, refuge-seeking bed bugs through the net. This assay was based on evidence that fully fed bed bugs rest near the host but are deterred from arresting (resting) on host-produced lipids [37]. Both bioassays used a two-jar system with a net “sandwiched” between the two jars (Fig. 1). The “net sandwich” was prepared by hollowing the solid lids of two plastic jars (5.5 cm × 4.8 cm each; Olcott Plastics, Saint Charles, IL, USA), securing a section of bed net between them with glue (Gorilla Glue, The Gorilla Glue Company, Cincinnati, OH, USA) and compressing it until it dried. The edges of the bed net cutting were trimmed and the joint sealed with stretched parafilm. The solid bottoms of both jars were replaced with tightly woven plankton netting (BioQuip Products, Rancho Dominguez, CA, USA) chemically bonded with dichloromethane. The top netting provided all life stages access to the blood, and both the top and bottom netting provided air circulation through the bioassay apparatus. Each of the two jars also contained a section of file folder (Manila) paper that ensured firm contact with the bed net and the plankton net (Fig. 2). These papers provided a walkway (ramp) that enabled unfed bed bugs to access the bed net, traverse it and be able to reach the feeder, whereas fed bed bugs could reach the bed net and cross it to rest in a dark aggregation site. After each bioassay, all jars were gently washed by hand with warm water and dish detergent.

### Bed net and bed bug dimensions

New bed nets were provided by the US Centers for Disease Control (CDC) and were selected as representative samples of untreated and commonly used and distributed insecticide-treated nets. The bed nets were (i) Siam Dutch, an untreated bed net (Siam Dutch Mosquito Netting Co., Bangkok, Thailand); (ii) Olyset Net (Sumitomo Chemical, Osaka, Japan), a permethrin-treated LLIN; (iii) PermaNet 2.0 (Vestergaard, Lausanne, Switzerland), a deltamethrin-treated LLIN (Table 1).

**Table 1:** Characteristics of bed nets used in this study, based on manufacturer specifications

LLIN name	Manufacturer	AI (g/kg)^a^	Denier^b^	Mesh size (holes/cm^2^)	Yarn type
Untreated	Siam Dutch	Not applicable	≥ 100	~ 24	Polyester multifilament
Olyset	Sumitomo Chemical	Permethrin (20)	≥ 150	~ 5.28	Polyethylene monofilament
PermaNet 2.0	Vestergaard	Deltamethrin (~ 1.6)	≥ 100	~ 24	Polyester multifilament

^a^Concentration of active ingredient (AI)

^b^A measurement of the weight of yarn (g) per 9000 m of the yarn used in net production

We empirically measured the length and width of 20 individual mesh holes in each bed net using an ocular micrometer in a microscope (Zeiss Stemi 47 50 57, Zeiss Microscopy, Jena, Germany). Additionally, we measured the width of the widest thoracic segment and widest point of the abdomen in both unfed and fully fed bed bugs. We measured 20 representative bed bugs of each life stage of both the Harlan and Fuller Mill strains using the same microscope used for the bed net measurements.

### Blood meal-seeking assay

Unfed bed bugs were placed in the bottom jar and challenged to cross the bed net to obtain a blood meal. Six or seven adult females were placed in the top jar of each assay to stimulate activation behavior. In each assay we used either a mixed stage cohort of 50 bed bugs consisting of 10 each of second, third and fifth instars, and adult males and females (*n* = 5 replicates), or a single cohort of 40 second instars (*n* = 5 replicates). All bed bugs were 10–14 days post feeding, which ensured that they were highly motivated to feed. Bed bugs were held in a 5.5 cm × 4.8-cm jar for up to 20 min in the dark at 20–40% relative humidity and 25–26 °C while the feeding apparatus was prepared. Each feeding membrane was gently dabbed three to four times on the forearm to transfer human body odor to the membrane. The jar holding the bed bugs was then attached to the bed net-sandwich assembly, and three to four breaths were exhaled into each top jar to activate bed bugs with CO_2_ and human odors. Each assay system was placed on a feeder for 1 h. All assays were conducted during the scotophase (nighttime), in a darkened room, at 20–40% relative humidity and 25–26 °C. The total number of blood-fed insects was recorded, representing the number of bed bugs that passed through the bed net. It is important to note that this assay did not account for bed bugs that might have crossed the net but failed to feed. Therefore, the assay recorded the minimum number of bed bugs that crossed the net. We also recorded the number of moribund (knocked down but able to respond to stimuli) and dead bed bugs (those no longer showing any response to stimuli) by gently touching bed bugs with forceps.

### Aggregation-seeking assay

In this assay, fresh fully fed bed bugs were placed in the top jar of the bioassay assembly and challenged to cross the bed net to reach a dark resting site. Newly molted second-instar bed bugs were starved 7–10 days and fed for 1 h in a 5.5 × 4.8-cm jar at 20–40% relative humidity and 25–26 °C without exposure to a bed net. Only fully fed bed bugs were selected and placed in a new jar that contained a fresh file folder paper walkway. This jar was connected as the top jar to the bed net-sandwich assembly. The bottom jar served as the target for aggregation-seeking fed bed bugs. To make this jar attractive to fed bed bugs, we “conditioned” file folder paper by exposing it to a colony of bed bugs, which impregnated it over time with feces and aggregation pheromone. A section of the aggregation-pheromone impregnated paper was placed within the target (bottom) jar, and the jar was wrapped completely in aluminum foil to create an attractive darkened aggregation site [38]. Furthermore, the whole bioassay assembly was placed horizontally in a 25 °C incubator on a photoperiod of 12:12 light:dark h at 35–45% relative humidity. The combination of the attractive paper and darkness served to attract bed bugs toward the aggregation jar, while the incubator lights repelled bed bugs away from the exposed starting jar. We counted and removed bed bugs from the target aggregation jar after 1 h, and then every 24 h, during the photophase (daytime) for 7 days. At each check, we removed the aggregation jar from the assay assembly, counted and removed the bed bugs (including dead bugs) and reconnected the aggregation jar to the bioassay system.

### Statistical analysis

All statistical analyses were conducted using SAS Enterprise Guide (v. 8.3, SAS Institute, Cary, NC), with *α* = 0.05. We compared the hole lengths (longer dimension) and widths (shorter dimension) of the three bed nets with separate one-way ANOVAs. The pre- and post-feeding thoracic and abdominal widths of bed bugs from both strains were compared using two-tailed paired Student’s *t*-tests. The thorax and abdomen widths of bed bugs (different life stages, different strains, unfed and fed) were compared to the mesh hole length of each bed net using one-way ANOVA followed by Dunnett’s multiple comparison tests, setting the bed net as the control to which bed bug sizes were compared. Results of the blood meal-seeking assays (proportion of bed bugs that traversed the net and blood-fed) were arcsine square root transformed and compared between the two strains and across the three bed nets using a two-way ANOVA. The results of the aggregation assays (proportion of bed bugs that traversed the net and aggregated in the dark jar over time) were arcsine square root transformed and compared using a two-way ANOVA. Mean time to aggregation was determined across bed net-strain pairs using survival analysis, and cumulative mortality on the bed nets was compared with the non-parametric Wilcoxon signed-rank test. All reported data were confirmed to be normally distributed, and measures of variation around the mean are standard errors (SE). Where box plots are shown, each represents the interquartile range (IQR, the difference between the third quartile and the first quartile), median, minimum and maximum values if there are no outliers or 1.5 times the inter-quartile range if outliers are present (represented by whiskers), outliers (if present) and mean (represented by *x*).

## Results

### Bed net and bed bug dimensions

**Fig. 3:**
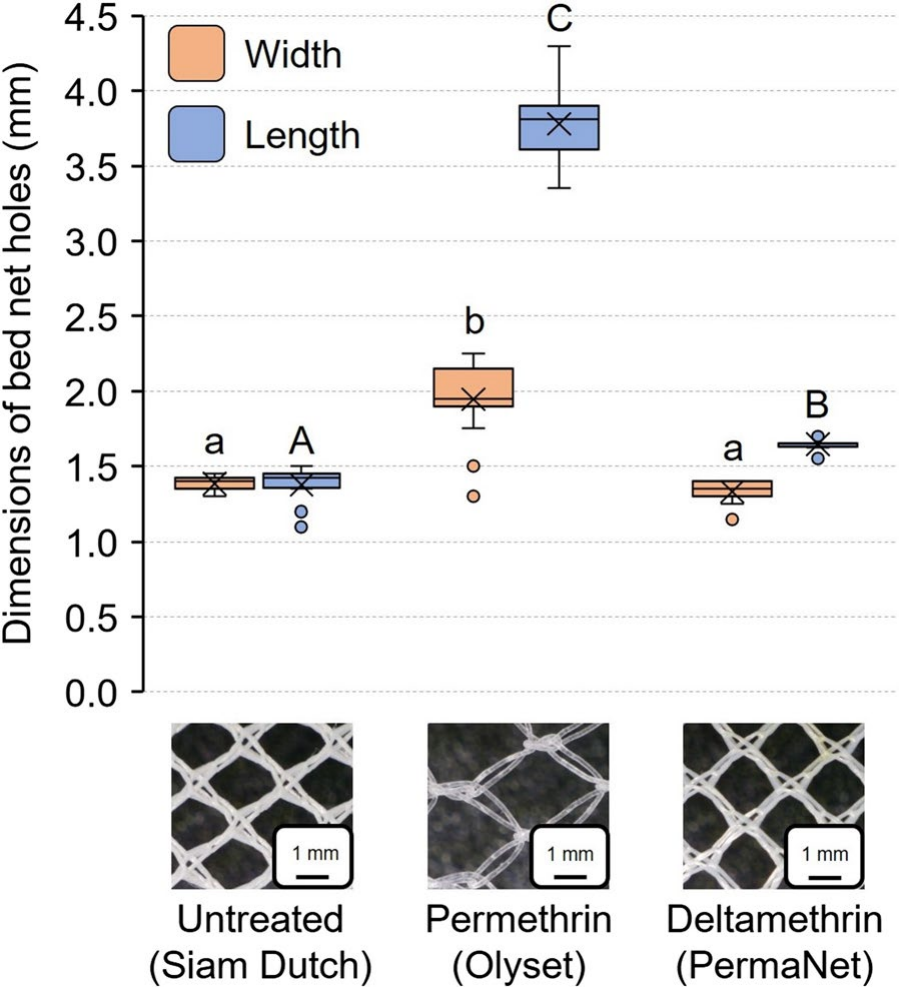
Comparisons of the dimensions of mesh holes in bed nets used in this study. The width (orange) and length (blue) of 20 randomly selected holes were measured for each of the three bed nets. The mean is represented by x within each box plot. Photos of each net are shown with the same scale bars (1 mm). The widths of bed nets that share lower case letters are not significantly different from each other, and the lengths of bed nets that share upper case letters are not significantly different from each other (ANOVA, Tukey’s HSD, *P* > 0.05)

The three bed nets we used differed in their insecticide treatment and the size of mesh holes through which bed bugs would need to pass. The untreated bed net (Siam Dutch) had relatively symmetrical holes measuring 1.39 ± 0.05 mm by 1.38 ± 0.13 mm (*n* = 20). The deltamethrin-treated bed net (PermaNet) was similar in hole dimensions to the untreated bed net (1.65 ± 0.04 mm by 1.33 ± 0.07 mm, *n* = 20). The permethrin-treated bed net (Olyset) had much larger oblong holes, measuring 3.78 ± 0.25 mm by 1.95 ± 0.24 mm (*n* = 20) (Fig. 3). Analysis of the nets revealed that hole lengths were significantly different across the three bed nets (*F* = 1333.07, *df* = 2, 57, *P* < 0.0001) (Fig. 3). Only the Olyset net had significantly wider holes than the other two nets (*F* = 102.91, *df* = 2, 57, *P* < 0.0001). Therefore, based on hole dimensions alone, we expected the bed nets with smaller holes to selectively allow only smaller bed bugs to pass through, whereas bed bugs should be least constrained passing through the Olyset net.

**Fig. 4:**
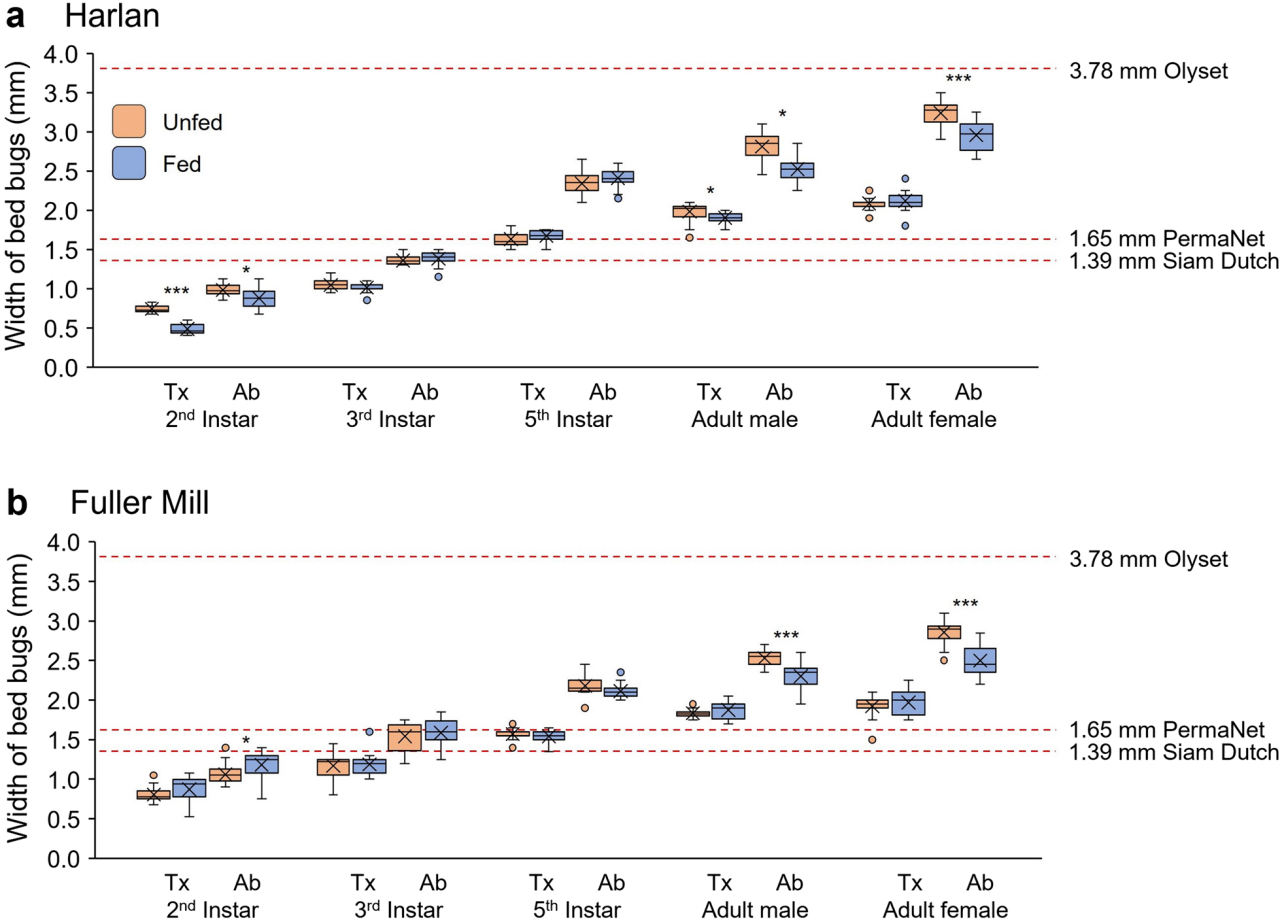
Comparisons of the thoracic and abdominal widths of unfed and fed *C. lectularius* of two strains. The thoracic (Tx) and abdominal (Ab) widths of second, third and fifth instars, as well as adult males and females (20 bed bugs per stage), were measured at both unfed (orange) and fed (blue) states for both the Harlan (insecticide-susceptible, **a**) and Fuller Mill (pyrethroid-resistant, **b**) strains. The results show increases in both thoracic and abdominal widths throughout development and general decreases in abdominal width in fed bed bugs. The mean is represented by x within each box plot. The average largest linear dimension of mesh holes in the three bed nets are represented by red dashed lines. Significant differences (Student’s *t*-test) in width between fed and unfed bed bugs are denoted by *P < 0.05; **P < 0.01; ***P < 0.001

We used a total of nearly 3800 bed bugs in the assay validation and data collection phases of this project. We measured the maximal thoracic and abdominal widths of various developmental stages of unfed and fed bed bugs of both strains (Fig. 4). Overall, the maximal thoracic and abdominal widths of fed bed bugs were similar to the respective widths of unfed bed bugs. In some stages (e.g. second instars, adults), fed bed bugs were significantly narrower than unfed bed bugs, indicating that the enormous increase in body mass associated with full engorgement on a blood meal resulted in longitudinal stretching of the abdomen and an increase in its girth that stretched the pleural membrane, decreasing the width of the abdomen. The mean thorax width of Harlan strain bed bugs ranged from 0.49 ± 0.01 mm in fed second instars to 2.12 ± 0.03 mm in fed adult females (*n* = 20 for each stage) (Fig. 4a) and in Fuller Mill bed bugs from 0.80 ± 0.02 mm in unfed second instars to 1.97 ± 0.03 mm in fed adult females (*n* = 20 for each stage) (Fig. 4b). Mean abdominal widths in Harlan bed bugs ranged from 0.88 ± 0.03 mm in fed second instars to 3.24 ± 0.03 mm in unfed adult females and in Fuller Mill bed bugs from 1.06 ± 0.03 mm in unfed second instars to 2.86 ± 0.03 mm in unfed adult females.

In general, the Harlan and Fuller Mill bed bugs broadly overlapped in their thoracic and abdominal widths. The thoracic widths of fed second instars and adult male Harlan bed bugs were significantly smaller post-feeding (Student’s *t*-tests, *t* = 13.16, *df* = 38, *P* < 0.001; *t* = 2.39, *df* = 38, *P* = 0.0219). Harlan second instars, as well as adult males and females, had significantly smaller abdominal widths after feeding (Student’s *t*-tests, *t* = 3.10, *df* = 38, *P* = 0.0042; *t* = 5.46, *df* = 38, *P* < 0.001; *t* = 5.17, *df* = 38, *P* < 0.001). Interestingly, the width of the thorax in Fuller Mill bed bugs did not change significantly in any life stage, whereas abdominal widths were significantly smaller in fed than unfed adult males and adult females (Student’s *t*-tests, *t* = 5.11, *df* = 38, *P* < 0.001; *t* = 6.36, *df* = 38, *P* < 0.001); the reverse was evident in second instars, with larger abdominal widths in fed nymphs (Student’s *t*-test, *t* = 2.50, *df* = 38, *P* = 0.018).

To estimate which bed bug stages might be excluded by each of the three bed nets, we statistically compared the maximum widths of each stage of unfed and fed bed bugs, respectively, with the hole lengths of each bed net. Resulting significant differences in size were associated with either a predicted ability to pass through the respective net (smaller size) or a predicted exclusion by the net (larger size), independently of the effects of insecticides. For brevity, only those life stages around the “cut-off” point, where exclusion or passage may be based on feeding status, as determined by statistical comparison, are presented. In the case of the untreated (Siam Dutch) bed net, we found that only second instars of both Harlan and Fuller Mill were significantly smaller than the measured hole length (*F* = 596.51, *df* = 40, 779, *P* < 0.0001; Dunnett’s test, *P* < 0.05), and therefore both fed and unfed second instars were predicted to pass through this net. Interestingly, most third instars of both strains were predicted to pass through unfed (Dunnett’s test, *P* < 0.05), but might potentially be excluded once fully fed (Dunnett’s test, *P* > 0.05) (Fig. 4). Similar results were seen with the deltamethrin-treated (PermaNet) net, as second instars of both strains, as well as third-instar Harlan bed bugs, were expected to pass regardless of feeding status (*F* = 606.38, *df* = 40, 779, *P* < 0.0001; Dunnett’s tests for both, *P* < 0.05). However, most third-instar Fuller Mill bed bugs, as well as most fifth instars of both strains, were predicted to be excluded once fully fed (all three Dunnett’s tests, *P* > 0.05) (Fig. 4). In the case of the permethrin-treated (Olyset) net, all measured life stages of both strains were significantly smaller than the mesh hole length, so most were predicted to pass through the net regardless of feeding status (*F* = 674.45, *df* = 40, 779, *P* < 0.0001; Dunnett’s tests for all, *P* < 0.05) (Fig. 4).

### Blood meal-seeking assays with multi-stage bed bug cohorts

**Fig. 5:**
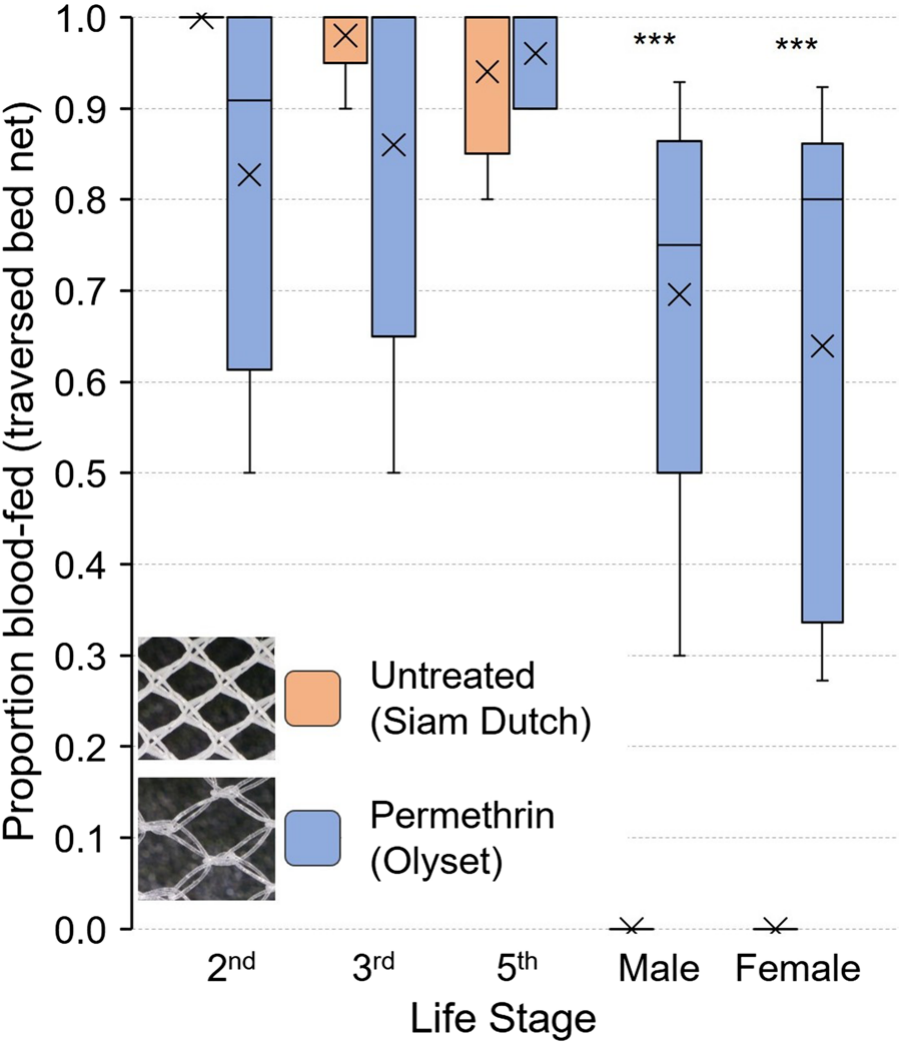
Comparison of the blood meal-seeking assays of insecticide-susceptible Harlan strain *C. lectularius* bed bugs of various life stages through the Siam Dutch untreated bed net and the Olyset permethrin-treated bed net. The mean is represented by x within each box plot. No significant differences were seen between the two bed nets in the proportion fed (traversed the bed net) for second, third or fifth instars. However, significantly more males and females passed through the permethrin-treated bed net (larger mesh holes) than through the untreated bed net (Student’s *t*-test, ***P < 0.0001)

To empirically investigate the predictions of statistical tests, we assayed mixed cohorts of Harlan strain bed bugs of all previously measured life stages to identify which would pass through the nets and which would not. We compared the untreated Siam Dutch bed net, with small mesh holes and no interference from insecticides, and the permethrin-treated Olyset bed net, with large mesh holes. In the blood meal-seeking assays, second, third and fifth instars readily passed through the untreated bed net, with mean feeding percentages of 100%, 98% and 94%, respectively, whereas 82.7%, 86% and 96% passed through the permethrin-treated bed net and blood fed (Fig. 5). Due to the large variation among replicates with the permethrin-treated bed net, we found no significant differences in the responses of second, third and fifth instars on the untreated and permethrin-treated bed nets (*P* > 0.05), which generally aligns with our statistical analysis of net hole length and bed bug size. Of note, however, is that while the passage of second and third-instar unfed bed bugs through the untreated net was expected based on our prior analysis, the passage of unfed fifth instars was not. This potentially relates to the pliable nature of the nets, which in certain cases might allow bed bugs to pass through successfully, as well as the more pliable nature of the bed bug abdomen than the thorax.

However, the untreated net completely excluded adult males and females from reaching the blood source (0% fed), whereas on average 69.6% and 63.9% of the males and females, respectively, traversed the permethrin-treated bed net and fed (Fig. 5). The two-way ANOVA analysis showed a significant effect of the overall model on bed bug blood feeding. Bed bug life stage, LLIN used and the interaction between stage and net significantly influenced the proportion of successfully blood-fed insects (Table 2). Specifically, the passage and feeding of adult bed bugs through the two nets were significantly different (Fig. 5) and aligned with the predicted results from our prior analysis. These results suggest that while bed nets with tight mesh (small holes) may stretch, and bed bugs may alter the width of their abdomen, these adjustments are constrained, as all adults were prevented from traversing the untreated net. Conversely, and again in line with our prior analysis, the permethrin-treated bed net, with large holes, permitted all life stages to pass through, suggesting that the insecticide did not repel or deter unfed, host-seeking bed bugs.

**Table 2:** Two-way ANOVA results for blood meal-seeking assays

Experiment	Term	*df* (model, error)	*F*-value	*P*-value
Multi-stage blood meal-seeking assay	Model	9, 40	33.17	< 0.0001
	Stage	4	49.89	< 0.0001
	LLIN	1	20.80	< 0.0001
	LLIN*Stage	4	19.55	< 0.0001
Single-stage blood meal-seeking assay	Model	5, 24	13.55	< 0.0001
	Strain	1	4.95	0.0357
	LLIN	2	24.15	< 0.0001
	LLIN*Strain	2	7.24	0.0035

### Blood meal-seeking assays with second instars of two bed bug strains

**Fig. 6:**
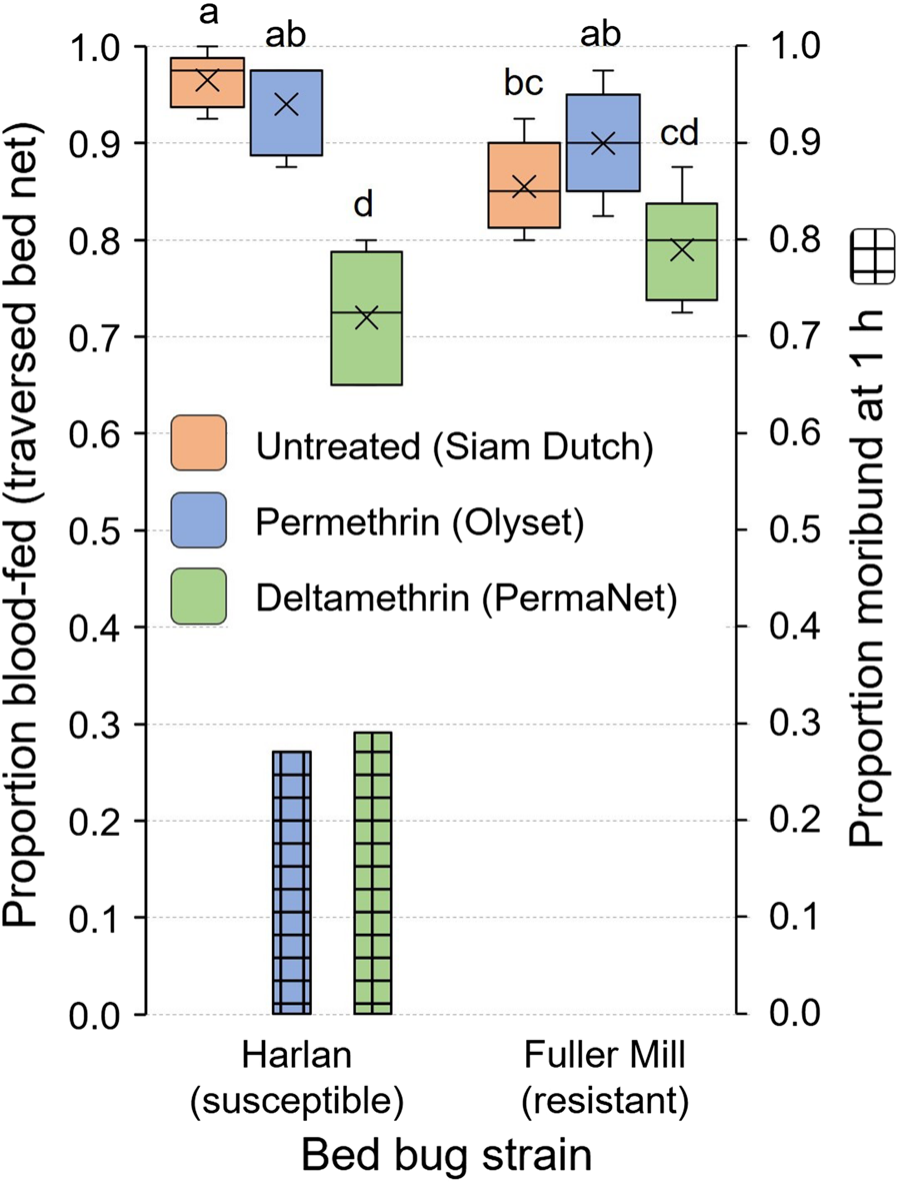
Comparison of the blood meal-seeking assays and mortality of second-instar *C. lectularius* bed bugs belonging to the insecticide-susceptible Harlan strain and the highly pyrethroid-resistant (resistance ratio > 1000) Fuller Mill strain. Comparisons were made between the two strains, as well as within strains, to assess the impact of pyrethroid resistance on blood meal-seeking behavior. The mean is represented by x within each box plot. Treatments that share lower case letters are not significantly different from each other (ANOVA, Tukey’s HSD, *P* > 0.05). Significantly lower proportions of blood-fed bed bugs were observed when bed bugs had to traverse deltamethrin-treated (PermaNet) bed nets. At 1 h, some Harlan (susceptible) bed bugs were moribund in these assays, and all moribund bed bugs died at 48 h, whereas no morbidity or mortality was observed in Fuller Mill (resistant) bed bugs

Because 100% of second instars of the Harlan strain passed through the untreated control bed net (Fig. 5), we compared their responses to those of pyrethroid-resistant bed bugs (Fuller Mill) on all three bed nets. Overall, in all replicates with both strains, ≥ 65% of the nymphs traversed the three bed nets and blood fed (Fig. 6). The two-way ANOVA analysis showed a significant effect of the model on successful blood feeding. All factors assessed, bed bug strain, bed net and the interaction of strain and net significantly impacted blood feeding (Table 2). Throughout the assay Harlan strain bed bugs appeared to be more active than Fuller Mill bed bugs (CCH, personal observation), and a higher percentage of Harlan second instars (96.5%) than Fuller Mill nymphs (85.5%) passed through the untreated bed net and fed (Tukey’s HSD, *P* = 0.0006) (Fig. 6). The responses of both strains were similar on the permethrin-treated Olyset bed nets, and in line with our prior analysis of net hole length and unfed bed bug width, with 94.0% of Harlan bugs and 90.0% of Fuller Mill bugs feeding (Tukey’s HSD, *P* > 0.05). Compared to the untreated bed net, lower percentages of second instars of both strains passed through the deltamethrin-treated PermaNet bed nets and fed (Harlan: 72.0%, Tukey’s HSD, *P* < 0.0001; Fuller Mill: 79.0%, *P* = 0.0009) (Fig. 6). These findings contrast with the predictions based on our comparison of net hole length and bed bug width, suggesting that deltamethrin may impede passage through the PermaNet bed net. In support of this, we found no significant differences in passage through the untreated net and the permethrin-treated Olyset bed net (Tukey’s HSD, *P* = 0.2428).

There was no mortality of Harlan or Fuller Mill bed bugs in the 60-min assay with untreated nets. However, 27% and 29% of the Harlan second instars were moribund after the 60-min assays on permethrin- and deltamethrin-treated bed nets, respectively. In contrast, none of the pyrethroid-resistant Fuller Mill bed bugs experienced any morbidity.

### Aggregation-seeking assays

**Fig. 7:**
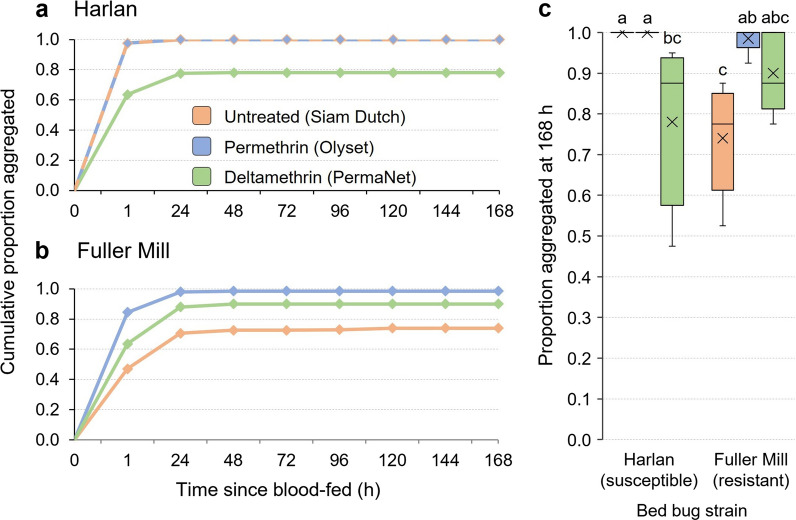
Comparison of cumulative time course of aggregation by second-instar *C. lectularius* bed bugs belonging to the insecticide-susceptible Harlan strain (**a**), and highly pyrethroid-resistant (resistance ratio > 1000) Fuller Mill strain (**b**). Assays were conducted with untreated (Siam Dutch, orange), permethrin-treated (Olyset, blue) and deltamethrin-treated (PermaNet, green) bed nets over the course of 7 days post-feeding or until all the bed bugs traversed the bed net into the aggregation jar. In **a**, the untreated and permethrin-treated nets followed the same aggregation pattern and so are represented by a dashed line of their respective colors. The majority of all observed aggregation occurred by 48 h for both strains, and Harlan bed bug aggregation was only impeded over the course of this assay by the deltamethrin-treated LLIN. Fuller Mill bed bugs never achieved 100% aggregation through any net, with the untreated net having the largest impeding effect. Statistical comparisons of the cumulative aggregation at 196 h (**c**). The mean is represented by x within each box plot. Treatments that share lower case letters are not significantly different from each other (ANOVA, Tukey’s HSD, *P* > 0.05). Significantly fewer Harlan bed bugs passed through the deltamethrin-treated LLIN and aggregated, and significantly fewer Fuller Mill bed bugs passed through the untreated net than the permethrin-treated LLIN

The aggregation-seeking assay was designed to simulate the passage of freshly fed bed bugs through a bed net in search of a refuge or aggregation site. We hypothesized that fed and unfed bed bugs would differ in their maneuverability through the three bed nets. This 7-day assay assessed bed bugs as they transitioned from a fully fed state to an unfed state. Across all three bed nets, and throughout the duration of the assay, all fully fed Harlan bed bugs crossed the untreated and permethrin-treated bed nets, and 100% were found in the aggregation jar within 24 h; < 80% crossed the deltamethrin-treated net during the 7-day assay (Fig. 7a). Some Fuller Mill bed bugs failed to cross all three bed nets even after 7 days; thus, < 100% were found in the aggregation jar by the end of the assay (Fig. 7b).

From survival analysis, we estimated the mean aggregation times (time to 50% aggregation) for the Harlan bed bugs as 1.6 ± 0.25, 1.6 ± 0.25 and 14.8 ± 1.38 h through the untreated, permethrin-treated and deltamethrin-treated bed nets, respectively (Fig. 7a). Correspondingly, mean aggregation times for the Fuller Mill nymphs were 33.6 ± 2.84, 5.0 ± 0.71 and 12.3 ± 1.16 h through the same bed nets (Fig. 7b). Cumulative aggregation for both strains across all three nets was compared at 1 h, 24 h and 168 h via two-way ANOVA. The model revealed a significant effect of the interaction between bed net and strain only at 1 h, and significant effects for both the net and strain predictor variables, as well as the interaction between bed net and strain, were seen at 24 and 168 h (Table 3). Overall, there was no significant effect of the permethrin-treated bed net on movement of the Harlan or Fuller Mill nymphs at any of the three time points (Tukey’s HSD tests, *P* = 0.18, *P* = 1.00, and *P* = 1.00, respectively), but a significant effect of the deltamethrin-treated bed net throughout (Tukey’s HSD, *P* = 0.0001 for all three time points).

**Table 3:** Two-way ANOVA results for aggregation-seeking assays at 1 h, 24 h and 168 h

Time points	Term	*df* (model, error)	*F*-value	*P*-value
1 h	Model	5, 24	2.71	0.0446
	Strain	1	3.23	0.0849
	LLIN	2	1.27	0.2993
	LLIN*Strain	2	3.89	0.0345
24 h	Model	5, 24	11.53	< 0.0001
	Strain	1	7.89	0.0097
	LLIN	2	11.724	0.0003
	LLIN*Strain	2	13.17	0.0001
168 h	Model	5, 24	10.64	< 0.0001
	Strain	1	4.56	0.0431
	LLIN	2	10.98	0.0004
	LLIN*Strain	2	13.33	0.0001

Across all three nets the final cumulative aggregation of fed second-instar bed bugs ranged from 47.5% (PermaNet) to 100% (Siam Dutch, Olyset) in Harlan and from 52.5% (Siam Dutch) to 98.5% (Olyset) in Fuller Mill (Fig. 7c). The two-way ANOVA of aggregation at 168 h revealed a significant effect of the overall model on bed bug aggregation (*F* = 10.64, *df* = 5, 24, *P* < 0.001). In fact, bed bug strain, LLIN used and the interaction of strain and LLIN all significantly impacted 168 h aggregation (Table 3). As expected from our prior comparison of net hole length and bed bug width, we found no significant effect of the untreated or permethrin-treated bed net on the 168 h aggregation of Harlan bed bugs (Tukey’s HSD, *P* = 1.00) (Fig. 7c). However, significantly fewer Harlan nymphs had traversed the deltamethrin-treated net by 168 h (Tukey’s HSD, *P* = 0.0001), once again suggesting the potential role of a barrier beyond hole size, likely the presence of deltamethrin. In line with our prior statistical comparisons yet again, we found no significant effect of the permethrin- or deltamethrin-treated bed nets on aggregation of Fuller Mill bed bugs at 168 h (Tukey’s HSD, *P* = 1.00), but unexpectedly, significantly fewer Fuller Mill nymphs traversed the untreated bed net throughout the assay compared to the permethrin-treated bed net (Tukey’s HSD, *P* = 0.0032) (Fig. 7).

**Fig. 8:**
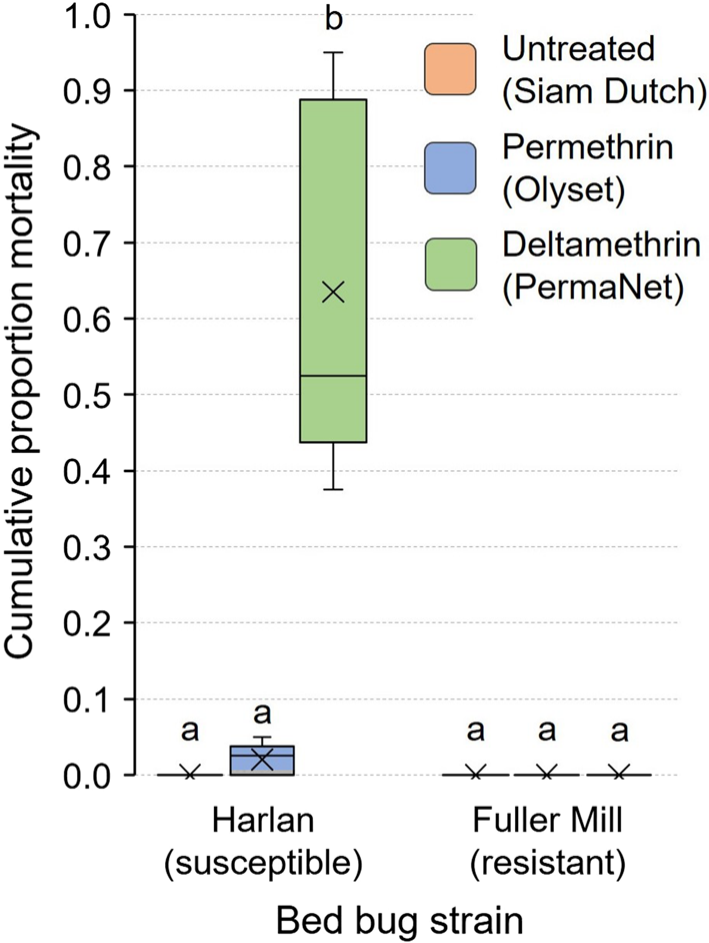
Cumulative 7-day mortality of second-instar *C. lectularius* bed bugs in the aggregation-seeking assay. Fully fed insecticide-susceptible Harlan strain bed bugs and the highly pyrethroid-resistant (resistance ratio > 1000) Fuller Mill bed bugs were challenged to pass through three different bed nets to reach an aggregation site. The mean is represented by an x within each box plot. Significantly higher mortality in Harlan bed bugs was seen only when they interacted with the deltamethrin-treated LLIN (Wilcoxon signed-rank test, **P = 0.0079)

Movement through the untreated bed net toward the aggregation jar caused no mortality in bed bugs of either strain during the 7-day assay (Fig. 8). However, interactions of fully fed Harlan strain nymphs with the permethrin- and deltamethrin-treated bed nets resulted in 2.0 ± 0.9% and 63.5 ± 10.7% mortality, respectively. There was no mortality in the resistant Fuller Mill bed bugs on these two bed nets. Thus, there was no significant difference in mortality of the Harlan and Fuller Mill bed bugs when interacting with the permethrin-treated bed net (Wilcoxon signed-rank test, *Z* = 1.81, *df* = 1, *P* = 0.1667). However, the Harlan nymphs experienced significantly higher mortality than the Fuller Mill bed bugs on deltamethrin-treated bed nets (Wilcoxon signed-rank test, *Z* = 2.67, *df* = 1, *P* = 0.0079) (Fig. 8).

## Discussion

To our knowledge, this is the first study to quantify the passage of bed bugs through commonly used LLINs in two distinct contexts, host-seeking by unfed bed bugs and shelter-seeking by fed bed bugs. Using innovative behavioral assays, we were able to show that the presence of commercial grade LLINs impregnated with permethrin or deltamethrin had no effect on these two critical bed bug behaviors in a highly resistant strain and only low, but varying effects on a highly susceptible strain which is unlikely to be seen in the field. Thus, our findings suggest that complete bed bug control is unlikely to be an ancillary benefit of current LLIN use. Instead, a large proportion of unfed bed bugs would be expected to readily pass through these bed nets to obtain blood meals from sleeping human hosts. Likewise, a large proportion of fully fed bed bugs would readily pass through bed nets returning to shelter and aggregation sites. Only some of the highly insecticide-susceptible bed bugs succumbed to the LLINs, and no pyrethroid-resistant bed bugs died.

We suspected that passage of bed bugs through bed nets would be limited by the mesh (hole) sizes of various nets and repellency associated with the presence of pyrethroid insecticides. As expected, physical interactions of bed bugs with bed nets, namely the dimensions of net holes in relation to bed bug size, affected which life stages of bed bugs could pass through each bed net to obtain a blood meal or return to harborage. Surprisingly however, pyrethroid-impregnated bed nets provided marginal additional barriers, beyond the physical barriers, to prevent the passage of bed bugs through bed nets. Overall, our findings suggest that the high motivation of unfed bed bugs to orient toward a blood-meal host and high propensity of fed bed bugs to follow aggregation stimuli likely contribute to recurrent lethal and sublethal interactions with LLINs, which in turn would select for the evolution of pyrethroid resistance in bed bug populations.

### Interactions of bed bugs and humans with LLINs

Bed bugs tend to aggregate on or near the bed or other furniture where humans rest and sleep, although at high population densities they may be more broadly distributed and shelter farther away from the bed. In general, bed bugs do not linger on the host beyond the several minutes required to take a blood meal, in part repelled or deterred by triglycerides associated with human skin [37]. Unfortunately, the behavioral ecology of the association of bed bugs with LLINs has not been previously investigated. Additionally, human behavior is expected to influence the interaction of bed bugs with bed nets. Correct usage of bed nets requires that the mattress be fully enclosed within the bed net each night [39]. This practice would likely enclose some bed bugs that aggregate on the mattress within the net, thus minimizing their passage through the LLIN, but would serve as a potential barrier to other bed bugs on the bed frame and around the home. Therefore, these bed bugs would then need to pass through the bed net at least twice every 7–14 days throughout the life of each bed bug to obtain at least one blood meal before each of its five molting events and to support the maturation of each batch of eggs and sperm [36, 40].

Our results suggest that when LLINs are properly used, bed bugs may need to alter their behaviors to obtain a blood meal. For example, more tightly woven nets, such as the PermaNet bed net, would likely restrict the movements of larger nymphs and adults towards both a host and aggregation sites. Therefore, we suspect that these larger stages would likely become trapped and harbor within the bed net due to their inability to leave once fully fed. Conversely, smaller bed bugs could readily transit through the bed net and could aggregate in safer locations away from the host. Notably, the much larger mesh holes of the Olyset net would allow all bed bug life stages to readily pass through the bed net.

In common usage, however, bed nets are frequently not fully tucked under the mattress and may dangle, even touching the bed frame and floor. In this case, the bed net may not serve as an effective barrier between the host and bed bugs. Instead, the LLIN may represent a walkway, connecting bed bugs from remote aggregations around the home to the host, independently of whether passage through the bed net is required or not. Under both scenarios, we envisage frequent interactions of bed bugs with LLINs, which are expected to select for pyrethroid resistance in the bed bug population. Further research is needed to understand how LLINs are used in the field, whether people change their use of LLINs when bed bugs proliferate and how LLINs with different mesh hole dimensions active ingredients or the incorporation of insecticide synergists modify the foraging behavior of bed bugs.

### Pyrethroid resistance in bed bugs and mosquitoes

The parallel evolution of adaptive responses to insecticides in mosquito disease vectors and bed bugs is not new. DDT, an organochlorine that shares the voltage-gated sodium channel target site with pyrethroids, has been widely used in vector control on a global scale [41]. DDT selected for various resistance mechanisms in both mosquitoes and bed bugs and preadapted both for resistance to more recent use of pyrethroids, including in outdoor sprays (for mosquitoes, but not bed bugs), IRSs and LLINs. Recent research has shown that exposure of *Aedes* and *Anopheles* populations to DDT has preselected for the evolution of multiple pyrethroid resistance mechanisms, including mutations that reduce target site sensitivity to the insecticide and metabolic detoxification [42–44]; similar mechanisms are likely to evolve in bed bug populations in the same communities. Together with the behavioral results presented here, these patterns strongly suggest that frequent exposure to pyrethroids in vector control programs likely would select for pyrethroid resistance in bed bugs.

We posit that unintentional selection on bed bugs might be more intense than on target mosquitoes for four main reasons. First, all life stages of the hemimetabolous bed bug are exposed to pyrethroids, whereas only adults, and mainly adult female mosquitoes are targeted indoors. Second, mosquitoes are often repelled before settling on LLINs due to the contact and potential spatial repellency of LLINs as well as the presence of other spatially repellent vector control tools, including mosquito coils [45]. Indeed, some LLINs (e.g. Olyset) are designed with large enough mesh holes that mosquitoes could pass through, but changes in mosquito behavior associated with repellency effectively prevent mosquitoes from doing so [46]. Therefore, direct and prolonged contact of mosquitoes with LLINs appears to be minimal. While mosquitoes may evolve olfactory adaptations that drive adaptive behavioral polymorphisms, leading to loss of LLIN repellency, our results suggest that bed bugs are unrepelled or minimally repelled by pyrethroids in LLINs, regardless of resistance status [47]. While some insecticide-susceptible bed bugs were killed during their passage through LLINs, many survived, and none of the pyrethroid-resistant bed bugs died, suggesting that LLINs would impose strong selection for resistance to pyrethroid insecticides. It is important to note that, to our knowledge, there are no pyrethroid-susceptible bed bug populations outside of laboratory colonies. Therefore, field populations of bed bugs are expected to more readily survive the brief interactions with LLINs that were imposed in our bioassays.

Third, related to the previous argument, adult mosquitoes fly and are relatively short-lived. In response to repellents, females may readily fly away and may even egress the home in search of a host. In contrast, bed bugs are long-lived, wingless and live strictly indoors. Therefore, bed bugs must find a blood meal within the home. Even if repelled by LLINs, bed bugs would remain in the home, capable of withstanding long periods of starvation, while searching for gaps in the LLIN-protection of the host. Finally, all these bed bug traits—dependence of all life stages on blood, wingless adults, low mobility, long-lived and resilience to starvation—contrast with mosquito traits and drive dramatically different population genetic structures in both insect taxa. Bed bug populations are highly inbred, often starting from small propagules of genetically related individuals [19]. Strong selection by LLINs and IRSs, especially on DDT-preadapted bed bugs, would quickly fix resistance alleles within a home population, eliminating reservoirs of susceptible individuals. This progression toward pyrethroid resistance would likely advance even in the face of fitness costs associated with specific resistance traits. In contrast, large outdoor reservoirs of insecticide-susceptible mosquitoes, coupled with high mobility of adult females, would slow the evolution of pyrethroid resistance, especially if resistance mechanisms incur significant fitness costs. Overall, we suggest that LLINs and IRSs, which are designed to target mosquitoes, likely impose much stronger selection for the evolution of pyrethroid resistance in bed bugs than in their intended targets.

### Constraints, limitations and pertinence to C. hemipterus

This investigation represents a narrowly focused study in the laboratory, meant to generate hypotheses that can be further tested in the field within LLIN-based malaria interventions. Therefore, we used small sections of bed nets to assess the minimal interactions of bed bugs with bed nets. We would expect that large bed bugs that attempt to pass through a LLIN with small mesh holes will likely walk on the net for much longer time than in our behavioral assays, and thus they would be exposed to much higher doses of insecticides. As mentioned already, although our bioassays assessed bed bug passage through bed nets in two contexts—host-seeking to blood feed and aggregation-seeking—we made no assumptions about the frequency of these behaviors during the months-long lifetime of bed bugs.

We evaluated the responses of only two populations of *C. lectularius* representing two extreme phenotypes—a highly insecticide-susceptible reference strain that has been reared in a laboratory setting for 5 decades, free of exposure to insecticides, and a highly pyrethroid-resistant strain. It is unlikely that highly insecticide-susceptible bed bug populations exist outside laboratory settings because of global widespread pyrethroid use and documented resistance [31, 48]. While the highly resistant strain is typical of bed bugs collected globally in residential settings, the magnitude and distribution of pyrethroid resistance in remote villages in malaria-endemic regions are yet to be thoroughly documented. Additionally, intraspecies level differences in host-seeking, feeding, aggregation behavior and mechanisms of insecticide resistance could arise in bed bug populations based on local selection and adaptations. Evolutionary trade-offs between insecticide resistance and various life history traits, including behaviors, have been documented in many insect species, including bed bugs [49, 50]. It is possible that lower feeding and aggregation responses, as we observed in the resistant bed bugs, represent such adaptive trade-offs that minimize movement and interaction with pesticides.

There is broad overlap in the distribution and incidence of *C. lectularius* and *C. hemipterus* in malaria-endemic regions. While our findings broadly apply to *C. hemipterus*, this species possesses unique morphological and behavioral characteristics that might lessen the barriers offered by LLINs and exacerbate the adverse interactions of humans with bed bugs within LLINs. *Cimex hemipterus* bed bugs are more adept climbers than *C. lectularius* and are overall smaller at each life stage [51, 52]. Unlike *C. lectularius* in the northern hemisphere, present day exposure of *C. hemipterus* to both DDT and pyrethroids likely continues to select for high resistance to the most common active ingredients used in LLINs.

## Conclusion: potential burden of bed bugs on malaria control

Our research demonstrated that new commonly used LLINs failed to prevent unfed bed bugs from passing through bed nets to obtain a blood meal and fed bed bugs from passing through in response to aggregation stimuli. Repeated lethal and sub-lethal exposure of bed bugs to LLINs would rapidly eliminate susceptible bed bugs and favor individuals with emergent resistance mechanisms. These results suggest that despite proper use of LLINs, pyrethroid-resistant bed bugs would proliferate. Thus, our findings support the concerns of recent research in sub-Saharan Africa, where the presence of pyrethroid-resistant bed bugs leads to LLIN abandonment, misuse and failure to regularly reimpregnate the bed net with insecticide [24, 26, 27, 30]. Recent research has sought to address issues of stalled progress in the fight against malaria, with a focus on bio-efficacy and a perspective beyond insecticide resistance [53, 54]. We propose that insecticide-resistant secondary pest populations, namely bed bugs, should be investigated as an additional potential factor contributing to stalled malaria control programs. Our findings also underscore the urgent need to invest in, reevaluate and innovate new designs of LLINs with a shared goal of effective mosquito and bed bug control.

## Data Availability

The datasets used and/or analyzed during the current study are available from the corresponding author on reasonable request.

## Abbreviations

AI: Active ingredient
DDT: Dichlorodiphenyltrichloroethane
IRS: Indoor residual spray
LLIN: Long lasting insecticide-treated net
VBD: Vector borne diseases
WHO: World Health Organization

## References

[1] Golding N, Wilson AL, Moyes CL, Cano J, Pigott DM, Velayudhan R (2015). Integrating vector control across diseases. BMC Med.

[2] Liu Q, Jing W, Kang L, Liu J, Liu M (2021). Trends of the global, regional and national incidence of malaria in 204 countries from 1990 to 2019 and implications for malaria prevention. J Travel Med.

[3] Hay SI, Guerra CA, Tatem AJ, Noor AM, Snow RW (2004). The global distribution and population at risk of malaria: past, present, and future. Lancet Infect Dis.

[4] Dhiman S (2019). Are malaria elimination efforts on right track? An analysis of gains achieved and challenges ahead. Infect Dis Poverty.

[5] Cibulskis RE, Aregawi M, Williams R, Otten M, Dye C (2011). Worldwide incidence of malaria in 2009: estimates, time trends, and a critique of methods. PLoS Med.

[6] Hemingway J, Shretta R, Wells TNC, Bell D, Djimdé AA, Achee N (2016). Tools and strategies for malaria control and elimination: what do we need to achieve a grand convergence in malaria?. PLoS Biol.

[7] Wilson AL, Courtenay O, Kelly-Hope LA, Scott TW, Takken W, Torr SJ (2020). The importance of vector control for the control and elimination of vector-borne diseases. PLoS Negl Trop Dis.

[8] Hemingway J (2014). The role of vector control in stopping the transmission of malaria: threats and opportunities. Philos Trans R Soc Lond B Biol Sci.

[9] Shretta R, Liu J, Cotter C, Holmes KK, Bertozzi S, Bloom BR (2017). Malaria elimination and eradication. Major infectious diseases.

[10] Tangena J-AA, Hendriks CMJ, Devine M, Tammaro M, Trett AE, Williams I (2020). Indoor residual spraying for malaria control in sub-Saharan Africa 1997 to 2017: an adjusted retrospective analysis. Malar J.

[11] Karunamoorthi K (2011). Vector control: a cornerstone in the malaria elimination campaign. Clin Microbiol Infect.

[12] Usinger RL (1966). Monograph of cimicidae.

[13] Harlan HJ, Faulde MK, Baumann GJ, Bonnefoy X, Kampen H, Sweeney K (2008). Bedbugs. Public health significance of urban pests.

[14] Gbakima AA, Terry BC, Kanja F, Kortequee S, Dukuley I, Sahr F (2002). High prevalence of bedbugs *Cimex hemipterus* and *Cimex lectularius* in camps for internally displaced persons in Freetown, Sierra Leone: a pilot humanitarian investigation. West Afr J Med.

[15] Suwannayod S, Chanbang Y, Buranapanichpan S (2010). The life cycle and effectiveness of insecticides against the bed bugs of Thailand. Southeast Asian J Trop Med Public Health.

[16] Dang K, Lilly DG, Bu W, Doggett SL (2015). Simple, rapid and cost-effective technique for the detection of pyrethroid resistance in bed bugs, *Cimex* spp. (Hemiptera: Cimicidae). Austral Entomol.

[17] Campbell BE, Koehler PG, Buss LJ, Baldwin RW (2016). Recent documentation of the tropical bed bug (Hemiptera: Cimicidae) in Florida since the common bed bug resurgence. Fla Entomol.

[18] Chebbah D, Elissa N, Sereno D, Hamarsheh O, Marteau A, Jan J (2021). Bed bugs (Hemiptera: Cimicidae) population diversity and first record of *Cimex hemipterus* in Paris. Insects.

[19] Saenz VL, Booth W, Schal C, Vargo EL (2012). Genetic analysis of bed bug populations reveals small propagule size within individual infestations but high genetic diversity across infestations from the eastern United States. J Med Entomol.

[20] Doggett SL, Dwyer DE, Peñas PF, Russell RC (2012). Bed bugs: clinical relevance and control options. Clin Microbiol Rev.

[21] Devries Z, Santangelo R, Barbarin A, Schal C (2018). Histamine as an emergent indoor contaminant: accumulation and persistence in bed bug infested homes. PLoS ONE.

[22] Helitzer DL, Kendall C, Wirima JJ (1993). The role of ethnographic research in malaria control: an example from Malawi. Res Soc Health Care.

[23] Deku G, Combey R, Doggett SL, Mensah BA (2021). Assessment of tropical bed bug (Hemiptera: Cimicidae), infestations in Cape Coast, Ghana: household control practices and efficacy of commercial insecticides and long-lasting insecticidal nets against field bed bugs. J Med Entomol.

[24] Temu EA, Minjas JN, Shiff CJ, Majala A (1999). Bedbug control by permethrin-impregnated bednets in Tanzania. Med Vet Entomol.

[25] Gunasekaran K, Sahu SS, Jambulingam P, Das PK (2005). DDT indoor residual spray, still an effective tool to control *Anopheles fluviatilis*-transmitted *Plasmodium falciparum* malaria in India. Trop Med Int Health.

[26] Malede A, Aemero M, Gari SR, Kloos H, Alemu K (2019). Barriers of persistent long-lasting insecticidal nets utilization in villages around Lake Tana, Northwest Ethiopia: a qualitative study. BMC Public Health.

[27] Parker W (2018). Community health priorities: lessons for malaria prevention from Balaka district. Malawi Malawi Med J.

[28] Tesfay K, Yohannes M, Mardu F, Berhe B, Negash H (2019). Assessment of community knowledge, practice, and determinants of malaria case households in the rural area of Raya Azebo district, Northern Ethiopia, 2017. PLoS ONE.

[29] Fourie J, Crafford D, Doggett SL, Miller DM, Lee C-Y (2018). The bed bug resurgence in Africa. Advances in the biology and management of modern bed bugs.

[30] Ingabire CM, Rulisa A, Van Kempen L, Muvunyi C, Koenraadt CJ, Van Vugt M (2015). Factors impeding the acceptability and use of malaria preventive measures: implications for malaria elimination in eastern Rwanda. Malar J.

[31] Dang K, Doggett SL, Veera Singham G, Lee C-Y (2017). Insecticide resistance and resistance mechanisms in bed bugs, Cimex spp. (Hemiptera: Cimicidae). Parasit Vectors.

[32] Johnson MS, Hill AJ (1948). Partial resistance of a strain of bed bugs to DDT residual. Med Newsl.

[33] Dang K, Doggett SL, Leong X-Y, Veera Singham G, Lee C-Y (2021). Multiple mechanisms conferring broad-spectrum insecticide resistance in the tropical bed bug (Hemiptera: Cimicidae). J Econ Entomol.

[34] González-Morales MA, DeVries Z, Sierras A, Santangelo RG, Kakumanu ML, Schal C (2021). Resistance to fipronil in the common bed bug (Hemiptera: Cimicidae). J Med Entomol.

[35] González-Morales MA, Thomson AE, Petritz OA, Crespo R, Haija A, Santangelo RG (2022). Systemic veterinary drugs for control of the common bed bug, *Cimex lectularius*, in poultry farms. Parasit Vectors.

[36] Saveer AM, DeVries ZC, Santangelo RG, Schal C (2021). Mating and starvation modulate feeding and host-seeking responses in female bed bugs, *Cimex lectularius*. Sci Rep.

[37] Gaire S, DeVries ZC, Mick R, Santangelo RG, Bottillo G, Camera E (2021). Human skin triglycerides prevent bed bug (*Cimex lectularius* L.) arrestment. Sci Rep.

[38] Gries R, Britton R, Holmes M, Zhai H, Draper J, Gries G (2015). Bed bug aggregation pheromone finally identified. Angew Chem Int Ed.

[39] World Health Organization. Instructions for treatment and use of insecticide-treated mosquito nets. 2002;4:1–51. http://apps.who.int/iris/bitstream/handle/10665/67573/WHO_CDS_RBM_2002.41.pdf;jsessionid=AD34D76BCB7B1241D3068A078AC2CB98?sequence=1.

[40] Matos YK, Osborne JA, Schal C (2017). Effects of cyclic feeding and starvation, mating, and sperm condition on egg production and fertility in the common bed bug (Hemiptera: Cimicidae). J Med Entomol.

[41] van den Berg H, Manuweera G, Konradsen F (2017). Global trends in the production and use of DDT for control of malaria and other vector-borne diseases. Malar J.

[42] Chen M, Du Y, Wu S, Nomura Y, Zhu G, Zhorov BS (2019). Molecular evidence of sequential evolution of DDT- and pyrethroid-resistant sodium channel in *Aedes aegypti*. PLoS Negl Trop Dis.

[43] Tchigossou G, Djouaka R, Akoton R, Riveron JM, Irving H, Atoyebi S (2018). Molecular basis of permethrin and DDT resistance in an *Anopheles funestus* population from Benin. Parasit Vectors.

[44] Silva JJ, Kouam CN, Scott JG (2021). Levels of cross-resistance to pyrethroids conferred by the *Vssc knockdown resistance* allele 410L + 1016I + 1534C in *Aedes aegypti*. PLoS Negl Trop Dis.

[45] Achee N, Masuoka P, Smith P, Martin N, Chareonviryiphap T, Polsomboon S (2012). Identifying the effective concentration for spatial repellency of the dengue vector *Aedes aegypti*. Parasit Vectors.

[46] Achee NL, Bangs MJ, Farlow R, Killeen GF, Lindsay S, Logan JG (2012). Spatial repellents: from discovery and development to evidence-based validation. Malar J.

[47] Kawada H, Ohashi K, Dida GO, Sonye G, Njenga SM, Mwandawiro C (2014). Insecticidal and repellent activities of pyrethroids to the three major pyrethroid-resistant malaria vectors in western Kenya. Parasit Vectors.

[48] Doggett SL, Feldlaufer MF, Doggett SL, Miller DM, Lee C-Y (2018). Limitations of bed bug management technologies. Advances in the biology and management of modern bed bugs.

[49] Homem RA, Buttery B, Richardson E, Tan Y, Field LM, Williamson MS (2020). Evolutionary trade-offs of insecticide resistance—the fitness costs associated with target-site mutations in the nAChR of *Drosophila melanogaster*. Mol Ecol.

[50] Gordon JR, Potter MF, Haynes KF (2015). Insecticide resistance in the bed bug comes with a cost. Sci Rep.

[51] Kim D-Y, Billen J, Doggett SL, Lee C-Y (2017). Differences in climbing ability of *Cimex lectularius* and *Cimex hemipterus* (Hemiptera: Cimicidae). J Econ Entomol.

[52] Deku G, Combey R, Doggett SL (2022). Morphometrics of the tropical bed bug (Hemiptera: Cimicidae) from Cape Coast. Ghana. J Med Entomol.

[53] Vinit R, Timinao L, Bubun N, Katusele M, Robinson LJ, Kaman P (2020). Decreased bioefficacy of long-lasting insecticidal nets and the resurgence of malaria in Papua New Guinea. Nat Comm.

[54] Lindsay SW, Thomas MB, Kleinschmidt I (2021). Threats to the effectiveness of insecticide-treated bednets for malaria control: thinking beyond insecticide resistance. Lancet Glob Health.

